# Supplementary material for: Neuromedin S Regulates Steroidogenesis through Maintaining Mitochondrial Morphology and Function via NMUR2 in Goat Ovarian Granulosa Cells
Source: Int J Mol Sci. 2022 Nov 2;23(21):13402. doi: 10.3390/ijms232113402 (PMC9655409; doi:10.3390/ijms232113402)
Supplement: Supplementary file 1 [file ijms-23-13402-s001.zip › ijms-1941876-supplementary.pdf]

Supplementary Materials

Neuromedin S Regulates Steroidogenesis through Maintaining Mitochondrial Morphology and Function via NMUR2 in Goat ovarian Granulosa Cells

Xuan Sun [1], Cheng Zeng [1,2], Feng Wang [1], Zhen Zhang [1], Fan Yang [1], Zhi-Peng Liu [1], Kang Li [1] and Guo-Min Zhang [1,2,*]

1 Jiangsu Livestock Embryo Engineering Laboratory, Nanjing Agricultural University, Nanjing 210095, China
2 College of veterinary medicine, Nanjing Agricultural University, Nanjing 210095, China
* Correspondence: zhangguomin@njau.edu.cn; Tel.: +86-025-84395381

Table S1. The nucleotide of amplified sequence and NCBI reference sequence blast results.

Amplified sequence genes	NCBI reference sequence Accession	Max Score	Total Score	Query Cover	Per. Ident	Acc. Len
NMS	XM_005686290.2	907	907	98%	99.20%	708
NMUR1	XM_005676958.2	2370	2370	100%	98.94%	1800
NMUR2	XM_005683202.3	1816	1816	99%	99.50%	2150

Table S2. Details of primer sequences, expected product sizes and Genebank accession numbers of gene cloning.

Items	Primer sequence (5′-3′)	Genebank No.	Fragment size (bp)
NMS	F: GGTTGTCTTTGCCGGTGGAA<br>R: ATTGCCTGATCCAGACTCGG	XM_005686290.2	577
NMUR1	F: GAGCGGATCCAGGAACCAAG<br>R: TTGGGGTGAAGCAGCATGAG	XM_005676958.2	1383
NMUR2	F: GTCTTGCATTTTAATAGCAGTTATGGA<br>R: AGAGTACAGAACCACACAGGGA	XM_005683202.3	1270

Table S3. Accession number of amino acid sequence.

Items	NMS Genebank No.	NMUR1Genebank No.	NMUR2 enebank No.
Goat	ACS32212.1	XP_005677015.2	XP_005683259.1
Cattle	NP_001070990.1	XP_002685694.3	NP_001030220.1
Pig	XP_020944053.1	NP_001302643.1	NP_001302544.1
Human	NP_001011717.1	AAH36543.1	NP_064552.3
Mouse	NP_001298717.1	AAI37776.1	AAI37612.1

Table S4. Details of siRNA sequences used for GCs transfection.

Gene	Sense (5'-3)	Antisense (5'-3')
NMUR1-goat-974	GGACACAGGUGACCAAGAUTT	AUCUUGGUCACCUGUGUCCTT
NMUR1-goat-774 (siNMUR1)	CCCGAGAGCCAUCUACAAATT	UUUGUAGAUGGCUCUCGGGTT
NMUR1-goat-1100	UCCAGUACGUGCACGUCAUTT	AUGACGUGCACGUACUGGATT
NMUR2-goat-650	GCAUCAAGCUCCACUACUUTT	AAGUAGUGGAGCUUGAUGCTT
NMUR2-goat-844 (siNMUR2)	GCAGAUAAAGUGACUGCAATT	UUGCAGUCACUUUAUCUGCTT
NMUR2-goat-844 (siNMUR2)	CCAGAAAGGAACUCUCAAATT	UUUGAGAGUUCCUUUCUGGTT
YAP1-goat-1817 (siYAP1)	CCGCCACCAAGCUAGAUAATT	UUAUCUAGCUUGGUGGCGGTT
Negative control	UUCUCCGAACGUGUCACGUTT	ACGUGACACGUUCGGAGAATT

Table S5. Details of primer sequences, expected product sizes and Genbank accession numbers of genes used for qPCR.

Items	Primer sequence (5′-3′)	Genebank No.	Fragment size (bp)
NMS	F: ATTGTGAAGTTTGAGCGGATGG<br>R: CTGCCAGACGCATTAGAGGG	XM_005686290.2	205
NMUR1	F: GAGCGGATCCAGGAACCAAG<br>R: AACAGAGAGGAACCTGAGCC	XM_005676958.2	210

NMUR2	F: GTGTTTGCTATCTGCTGGGC<br>R: GGTAGAAGAAGACACCTGACACC	XM_005683202.3	320
STAR	F: GGTCCCCGAGACTTTGTGAG<br>R: AATCCACTTGGGTCTGCGAG	XM_013975437.2	262
CYP11A1	F: CACTTTCGCCACATCGAGAAC<br>R: AGGCTCCTGACTTCTTAAACAGG	NM_001287574.1	217
3BHSD	F: AGACCAGAAGTTCGGGAGGAA<br>R: TCTCCCTGTAGGAGTTGGGC	XM_013962473.2	292
CYP19A1	F: TGGTGTCCGAAGTTGTGCCTATTG<br>R: AAGGTCGAACAGCTTTCCAGAGTG	XM_013967046.2	393
MST1 (STK4)	F: TCAGATCAACAGCTTCGGCA<br>R: ACCGTCCAGCTCTTCAGAAAC	XM_013968939.2	105
MST2 (STK3)	F: CAGCGCCCAAGAGTAAGCTA<br>R: GCCACAACTTGACCAGATTCC	XM_018058284.1	148
LATS1	F: CTCATCAGCAACGTCTACATCG<br>R: TCTTGAGATAATCCAACCCGCA	XM_018053335.1	70
LATS2	F: AGTGCTCCTCCGGAAAGGTTA<br>R: GCGTGCTCTCCCAGTTAATC	XM_018056548.1	146
YAP1	F: TGACCCTCGTTTTGCCATGA<br>R: TCTGTTGCTGCTGGTTGGAA	XM_018059884.1	128
MFN1	F: ATCAGGGAGGTTACAGAGGAGGTTG	XM_018047540.1	226

	R: GGGATTGAAGCATTGAAGCGTTGAC		
MFN2	F: GTTGTTGGAGGAGTGGTGTGGAAG<br>R: TCTGGTTCATGGCGGCGATTTC3	XM_018060333.1	301
OPA1	F: GAACGCAGCATTGTTACAGACTTGG<br>R: AGCCTGTTGTTCAACTGACTCTCG	XM_005675105.2	363
DNM1L	F: TTCCAATTATGCCAGCCAGTCCAC<br>R: GTGCCATGTCCTCAGATTCAGTCAG	XM_018048322.1	294
FIS1	F: CTGAACGAGTTGGTGTCTGTGGAG<br>R: GGTTGTTCTGCGGCTCTGTCTG	XM_005697811.3	304
ClpP	F: GGTGGAACAGACGGGTCG<br>R: GGGCTTCTTGTTGCTTTCCG	XM_018050927.1	157
PKR	F: CCAAGCAACCAAAGAACCAGC<br>R: CTTTGATGCCCCCTTTCCAGT	XM_018055124.1	179
JNK	F: ATGGGCTACAAAGAGAACGTTGATA<br>R: ATTCCTCACAGTTGGCTGAAGT	XM_018051622.1	177
JUN	F: GGAAACGACCTTCTACGACGATGC<br>R: CCGTTGCTGGACTGTATGATGAGG	XM_018044742.1	243
HSP60	F: CGTTGCTGTGTTGAAGGTTGG<br>R: AAGGCTGGAATGCACCGAAG	XM_018061271.1	153
GAPDH	F: CCGTTCGACAGATAGCCGTAA<br>R: CCGTTCGACAGATAGCCGTAA	XM_005680968.3	296

Table S6. Details of antibodies.

Antibodies name	Cat NO.	Source	Dilutions used in IHC	Dilutions used in WB	Dilutions used in IF
NMS antibody	DF4237	LTD (USA)	1:250	1:800	1:200
Anti-NMUR1 antibody	AF9133	LTD (USA)	1:250	1:1000	1:200
NMUR2 polyclonal antibody	bs-11421R	Bioss (Beijing, China)	1:250	1:500	1:200
StAR polyclonal antibody	bs-3570R	Bioss (Beijing, China)	–	1:1000	–
CYP11A1 polyclonal antibody	bs-10099R	Bioss (Beijing, China)	–	1:1000	–
Anti-HSD3B1 antibody	NB110-78644SS	Novus (USA)	–	1:200	–
Cytochrome P450 19A1 antibody	DF6884	LTD (USA)	–	1:2000	–
STK4/MST1 polyclonal antibody	22245-1-AP	ProteinTech (IL, USA)	1:200	1:2000	1:100
STK3/MST2 polyclonal antibody	12097-1-AP	ProteinTech (IL, USA)	1:200	1:2000	1:200

Anti-MST1/2(Phospho-Thr183) antibody	D155304	BBI (Shanghai, China)	–	1:500	–
LATS1 polyclonal antibody	17049-1-AP	ProteinTech (IL, USA)	1:200	1:2000	1:100
Anti-LATS2 antibody	D260914	Sangon Biotech (Shanghai, China)	1:200	1:1000	1:100
Anti-LATS1/2(Phospho-Ser909/872) antibody	D155305	BBI (Shanghai, China)	–	1:1000	–
YAP1 polyclonal antibody	13584-1-AP	ProteinTech (IL, USA)	1:200	1:2000	1:100
Anti-YAP1(Phospho-Ser127) antibody	D151452	BBI (Shanghai, China)	–	1:1000	–
PGC1 alpha polyclonal antibody	NBP1-04676SS	Novus (USA)	–	1:500	–
Phospho-PGC1α(S571) polyclonal antibody	AF6650-SP	R&D systems (USA)	–	1:1000	–
NFN1 polyclonal antibody	13798-1-AP	ProteinTech (IL, USA)	–	1:1000	–
NFN2 polyclonal antibody	12186-1-AP	ProteinTech (IL, USA)	–	1:4000	–
OPA1 polyclonal antibody	27733-1-AP	ProteinTech	–	1:2000	–

		(IL, USA)			
DRP1(C-terminal) polyclonal antibody	12957-1-AP	ProteinTech (IL, USA)	–	1:2000	–
FIS1 polyclonal antibody	10956-1-AP	ProteinTech (IL, USA)	–	1:2000	–
CLPP polyclonal antibody	15698-1-AP	ProteinTech (IL, USA)	–	1:4000	–
Anti-EIF2AK2(PKR) antibody	bs-1493R	Bioss (Beijing, China)	–	1:500	–
JNK1/2/3 polyclonal antibody	51153-1-AP	ProteinTech (IL, USA)	–	1:1000	–
Phospho-JNK1/2/3(Thr183+Tyr185) polyclonal antibody	AF3318	LTD (USA)	–	1:500	–
Phospho-JUN(Ser73) polyclonal antibody	28891-1-AP	ProteinTech (IL, USA)	–	1:1000	–
HSP60 antibody	AF5374	LTD (USA)	–	1:1000	–
GAPDH Mouse Monoclonal antibody	60004-1-Ig	ProteinTech (IL, USA)	–	1:8000	–

HRP-conjugated Affinipure Goat Anti-Rabbit IgG(H+L)	SA00001-2	ProteinTech (IL, USA)	–	1:5000	–
HRP-conjugated Affinipure Goat Anti-Mouse IgG(H+L)	SA00001-1	ProteinTech (IL, USA)	–	1:5000	–
Goat Anti- Rabbit IgG H&L (DyLight® 650)	ab96886	Abcam (IL, USA)	1:100	–	–

IHC: immunohistochemistry, WB: Western blot, IF: immunocytochemistry, (−): absent.

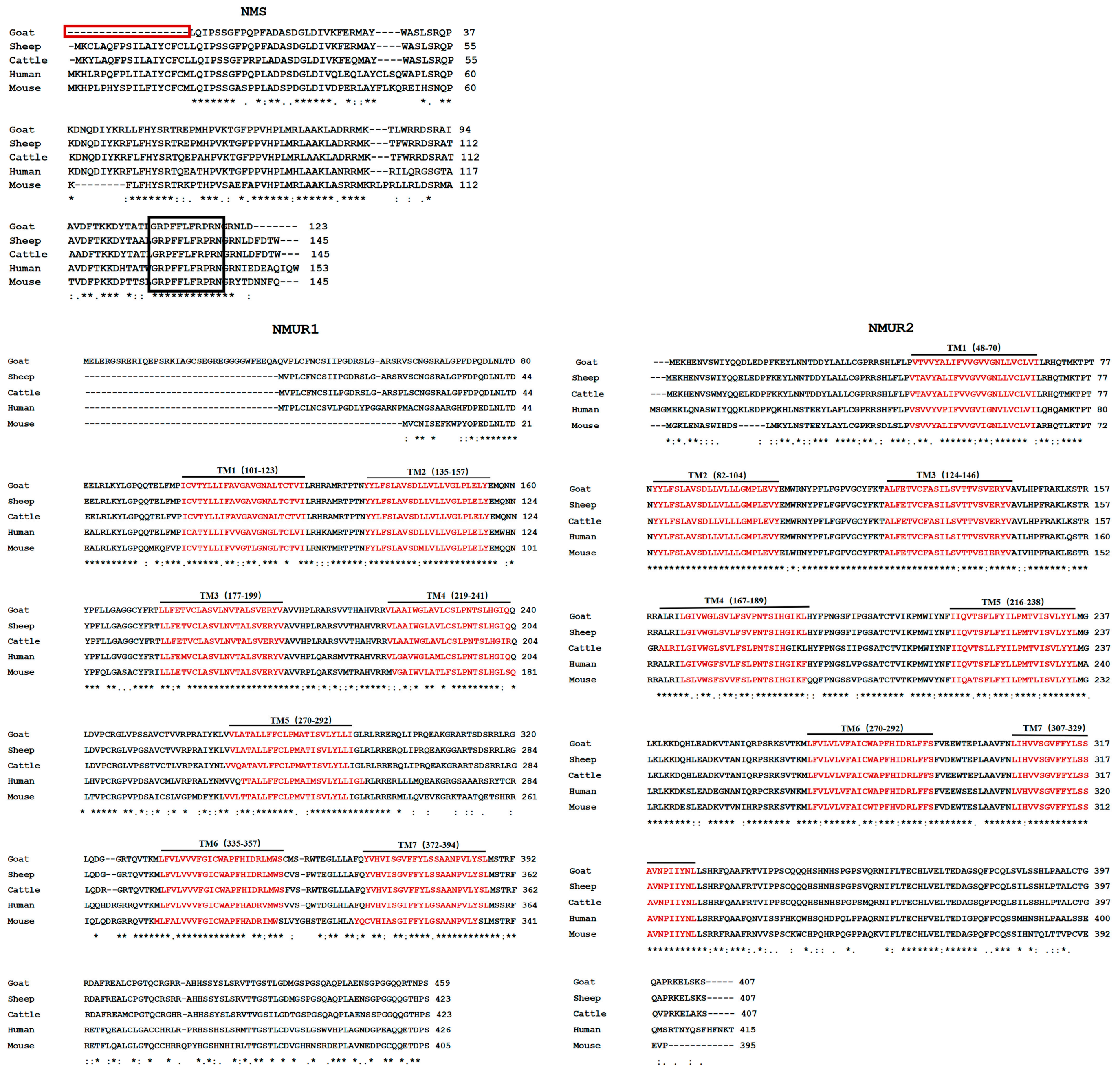

Figure S1. Analysis of goat NMS and its receptor amino-acid sequences. Multiple alignments of deduced amino-acid sequences of NMS and its receptor in several species. The lack of 19 amino acids of the goat NMS was shown in the red box, and the core structure of NMS binding with its receptors show in the black box. Transmembrane region of the goat NMUR1and NMUR2 amino-acid sequence fragment. 101-123aa, 135-157aa, 177-199aa, 219-241aa, 270-292aa, 335 - 357aa and 372-394aa, NMUR1 transmembrane sequence; 48-70aa,82-104aa, 124-146aa, 167-189aa, 216-238aa, 270 - 292aa and 307-329aa, NMUR2 transmembrane sequence. Identifiers: “*” identical, “.” and “:” conservative replacements, “” mismatches.

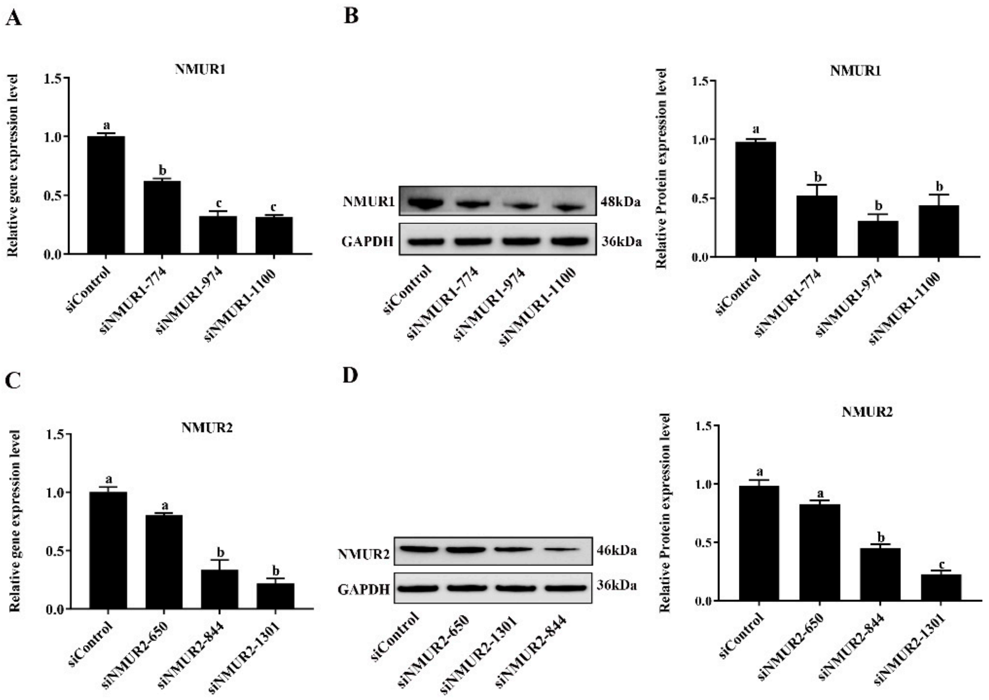

Figure S2. NMS receptors interference efficiency verification in goat granulosa cells. (A) The mRNA expression of NMUR1 in goat GCs under siNMUR1-774, siNMUR1-974, and siNMUR1-1100 treatment was determined by qPCR. (B) The protein expression of NMUR1 in goat CSs under siNMUR1-774, siNMUR1-974, and siNMUR1-1100 treatment was shown by western blot. (C) The mRNA expression of NMUR2 in goat GCs under siNMUR2-650, siNMUR2-844, and siNMUR2-1301 treatment was determined by qPCR. (D) The protein expression of NMUR2 in goat GCs under siNMUR2-650, siNMUR2-844, and siNMUR2-1301 treatment was shown by western blot. The data are expressed as fold change. Data are presented as mean ± S.E.M. of the results obtained in at least three independent experiments. Different letters indicate significant differences in the expressions between the groups ($P < 0.05$).

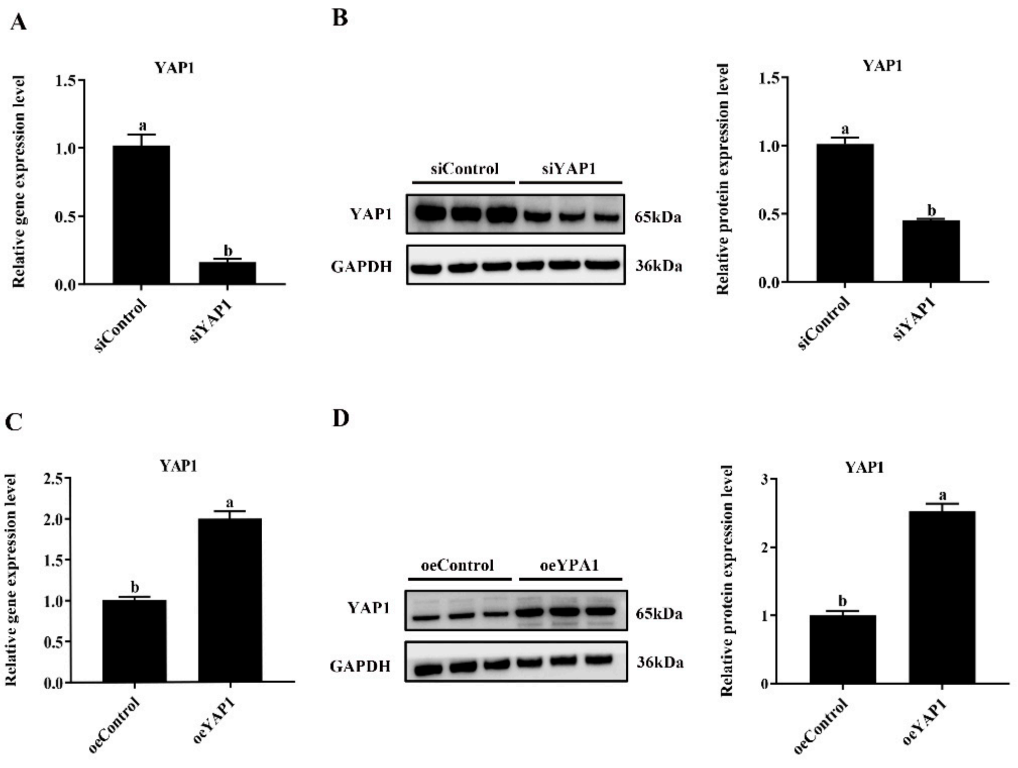

Figure S3. YAP1 interference and overexpression efficiency verification in goat granulosa cells. (A) The mRNA expression of YAP1 in goat GCs under siYAP1-1817 treatment was determined by qPCR. (B) The protein expression of YAP1 in goat GCs under siYAP1-1817 treatment was determined by western blot. (C) The mRNA expression of oeYAP1 treatment in goat GCs was determined by qPCR. (D) The protein expression of oeYAP1 treatment in goat GCs was determined by western blot. Data are presented as mean ± S.E.M. value from at least three individuals. Different letters indicate significant differences in the expressions between the groups ($P < 0.05$).
